# On the Indications of the Pulse

**Published:** 1831

**Authors:** 


### ON THE INDICATIONS OF THE PULSE. By DR. BURNE.

IN our contemporary of the Midland counties, for May last, Dr. Burne of Spring Gardens, has published some physiological observations on the motions and structure of the heart, which had been read at the Physical Society of Guy's Hospital, three years ago, and now sent forth on account of the discussions which are afloat respecting the motions and sounds of the heart. We apprehend that these discussions have led to no conclusive result, much less to any useful purpose. We shall therefore pass over the physiological portion of this paper, and give our readers the practical part with which the communication winds up.

INDICATIONS OF THE PULSE.

"The character of the pulse depends on three circumstances, namely, the volume of blood sent forth by the left ventricle at each contraction of the heart; the force with which that blood is propelled; and the degree of tonicity of the artery. These causes, together with the frequency of the heart's action, give rise to all the varieties of the pulse. The character of the pulse is, moreover, modified by the state of the skin, according as its temperature is high or low, or as it is dry and contracted, or relaxed and perspirable.

The volume of blood sent forth by the ventricle at each contraction, is necessarily regulated by many circumstances: as by the supply of blood which that ventricle receives, and by the area of the mouth of the aorta, &c. The supply of blood may be abundant, from a general fulness of the system, and activity of the circulation; or scanty, from an opposite state of things. The supply may also be deficient, from a diseased condition of the lungs opposing the transmission of blood from the right to the left side of the heart, or from a narrowing of the auriculo-ventricular opening. The area of the mouth of the aorta may be diminished by disease of its valves, or increased by dilatation. All these different circumstances influence the character of the pulse, and the pulse, therefore, becomes an indicative sign.

Notwithstanding the pulse is mainly produced by the action of the heart, it does not always correspond with that action. The action of the heart will sometimes be impetuous and strong, while the pulse is small and weak; as in a narrowing of the orifice of the mitral valve from cartilaginous or osseous deposit.

The various characters of the pulse may be represented by the following designations. It may be strong, weak, hard, soft, wiry, sharp, harsh, grating, jarring, vibrating, falling back, fleeting; or full, large, small, thready; or yielding, open, contracted, tight; or rapid, frequent, accelerated, slow; or it may be quick, free, equal, unequal, regular, irregular, hesitating, labouring, intermittent, fluttering.

Each of these terms designates some notable peculiarity of the pulse; and as they are numerous, and as most of them cannot be measured except by the sensation produced in the mind, it follows that the accuracy of this measurement must depend on the skill of individuals, and hence the difficulty of an acquaintance with the science of the pulse. But although the task is difficult, it may be accomplished by diligence and perseverance. The ready use of the stethoscope, requires the ear to be educated; and an education of the touch is necessary to a correct judgment of the pulse.

If time can be profitably spent in acquiring a knowledge of mediate auscultation, much more may it be so spent in acquiring a knowledge of the pulse; because the pulse is one of the most prominent signs in disease, and one of the most certain indications in the treatment. Without the assistance of the pulse, we cannot advantageously, or even safely, employ blood-letting, which is the most powerful of all our remedial agents, the most beneficial when judiciously prescribed, the most fatal when prescribed in error.

The characters of the pulse are produced by three causes; the heart, the volume of blood, and the artery; and as these causes always operate, it follows that every given pulse must have several characters. Thus, the same pulse may be strong, full, and firm; the strength resulting from the heart, the fulness from the volume of blood, and the firmness from the tonicity of the artery.

Of the terms already specified, some belong to the heart, some to the volume of blood, some to the artery, and some to these causes combined.

Those which depend upon the heart, are *strong*, *weak*, *sharp*, *jarring*, *rapid*, *frequent or accelerated*, *unfrequent*,

*quick, slow, equal, unequal, regular, irregular, hesitating, labouring, intermittent, fluttering.*

Those which depend upon the volume of blood, are *full* and *small.*

Those which depend upon the artery, are, *contracted, tight, yielding, open, harsh, grating.*

Those which depend upon the above causes combined, are *hard, soft, wiry, vibrating, falling back, fleeting, thready, large, compressible.*

The pulse, in health, beats about 72 strokes in the minute, or thereabout, and the number varies a little in the course of the day and night, being rather more than 72 in the evening, and less than 72 before rising in the morning. Its natural character is *equal, regular, soft,* and of *moderate strength and volume.*

In the deviations from health, the pulse varies in character according as the nervous or sanguiferous systems are prominently affected, or as both are affected simultaneously; and it is this difference which gives rise to that peculiarity of pulse, which surgeons meet with so frequently after the shock of an accident or operation, and which is known by the term *irritable pulse,* or *pulse of irritation.* This pulse derives its peculiarity from the excitement of the nervous system; and its character may be described as follows: it is frequent, the stroke is quick, short, rather sharp, but not strong, the impression on the finger being rather smart but not lasting. Its volume may be small or otherwise, but is never full.

There are many diseases and conditions of the body which are accompanied by a particular kind of pulse, and in some of which the peculiar pulse is in part diagnostic. As, for example, the pulse accompanying pneumonia, pleurisy, apoplexy, and depression of the scull from fracture; concussion of the brain, adhesion of the pericardium, valvular disease of the heart, and that succeeding sudden hæmorrhage, and therefore called the hæmorrhagic pulse. This last is of so much importance, as to deserve our best attention: for the efforts of the heart to keep up the circulation, after sudden and copious hæmorrhage, give a character to the pulse, which is apt to be regarded as indicative of increased power, and so to lead to error in practice.

The hæmorrhagic pulse is frequent and open, and the stroke is quick and rather smart, but short and falling back, and leaves but a slight impression on the finger. This open character is sometimes mistaken for a full pulse, and the quick and rather smart stroke construed into strength, which may tempt a practitioner to abstract blood, while the symptoms are already produced by the loss of blood: but the slight impression upon the finger, and the sensation of falling back after every stroke, will at once determine that there is a deficiency of strength and volume of blood.

The pulse of an intense pneumonia, is the best example which can be furnished of a full pulse, the very nature of the disease causing, in the early stage, a very abundant supply of blood to the left ventricle.

Pleurisy offers an example of a firm pulse; so also do some cases of concussion of the brain, during the succeeding inflammatory stage.

The adhesion of the pericardium is attended with so peculiar a pulse, as to be a diagnostic sign. It is generally said that there are no signs by which the adhesion of the pericardium can be detected; and this is true to a certain extent. It is true, for instance, where the adhesion is by the medium of a rare and loose cellular tissue which allows the free motion of the heart; then there are no signs, because the action of this organ is not interfered with; but where the adhesion is firm and close, it so impedes the action of the heart, as to give a decided character to the pulse. The pulse, under these circumstances, is frequent; the stroke is unpleasantly sharp; is quick and strong more or less, and more or less full.

In ascertaining the nature of the pulse, we must be circumspect, and take care that we are not led into error by any accident or idiosyncracy: for instance, any inflammation of the finger, or rheumatism of the wrist, will affect the pulse of the affected arm; or if one arm has been lying out of bed, while the other has been covered, the pulse in the two arms will differ exceedingly; or there may be a naturally vigorous stroke of the heart, which is usually attended with a thick strong artery, and so on.

The characters of the pulse which I have attempted to draw, will, I am afraid, be regarded by some as unnecessarily minute; and they may appear difficult to become acquainted with; but they will easily be made familiar by patience and industry; and the satisfactory, and, I may add, practical information, which I have gleaned, in proportion as I have devoted time and attention to the contemplation of the characters of the pulse, urge me to recommend the study of its indications, as leading to the acquisition of valuable knowledge."

